# Transcranial Near Infrared Light Stimulations Improve Cognition in Patients with Dementia

**DOI:** 10.14336/AD.2021.0229

**Published:** 2021-07-01

**Authors:** Damir Nizamutdinov, Xiaoming Qi, Marvin H Berman, Gordon Dougal, Samantha Dayawansa, Erxi Wu, S. Stephen Yi, Alan B Stevens, Jason H Huang

**Affiliations:** ^1^Baylor Scott and White Health, Neuroscience Institute, Neurosurgery, Temple, TX, USA.; ^2^Texas A&M University, HSC, College of Medicine, Neurosurgery, Temple, TX, USA.; ^3^Quietmind Foundation, Elkins Park, PA, USA.; ^4^Maculume Limited, Spennymoor, UK.; ^5^Texas A&M University, HSC, College of Pharmacy, Department of Pharmaceutical Sciences, College Station, TX, USA.; ^6^Department of Oncology, Dell Medical School, The University of Texas at Austin, TX, USA.

**Keywords:** transcranial near infrared light, photobiomodulation, dementia, tnir light treatment

## Abstract

Dementia is a complex syndrome with various presentations depending on the underlying pathologies. Low emission of transcranial near-infrared (tNIR) light can reach human brain parenchyma and be beneficial to a number of neurological and neurodegenerative disorders. We hereby examined the safety and potential therapeutic benefits of tNIR light stimulations in the treatment of dementia. Patients of mild to moderate dementia were randomized into active and sham treatment groups at 2:1 ratio. Active treatment consisted of low power tNIR light stimulations with an active photobiomodulation for 6 min twice daily during 8 consequent weeks. Sham treatment consisted of same treatment routine with a sham device. Neuropsychological battery was obtained before and after treatment. Analysis of variance (ANOVA) was used to analyze outcomes. Sixty subjects were enrolled. Fifty-seven subjects completed the study and had not reported health or adverse side effects during or after the treatment. Three subjects dropped out from trial for health issues unrelated to use of tNIR light treatment. Treatment with active device resulted in improvements of cognitive functions and changes were: an average increase of MMSE by 4.8 points; Logical Memory Tests I and II by ~3.0 points; Trail Making Tests A and B by ~24%; Boston Naming Test by ~9%; improvement of both Auditory Verbal Learning Tests in all subtest categories and overall time of performance. Many patients reported improved sleep after ~7 days of treatment. Caregivers noted that patients had less anxiety, improved mood, energy, and positive daily routine after ~14-21 days of treatment. The tNIR light treatments demonstrated safety and positive cognitive improvements in patients with dementia. Developed treatment protocol can be conveniently used at home. This study suggests that additional dementia treatment trials are warranted with a focus on mitigating caregivers’ burden with tNIR light treatment of dementia patients.

Dementia is a complex syndrome with different clinical presentations. Although the course of disease and its clinical progression vary largely contingent on the underlying pathologies, the development of brain dysfunction that typically progresses to diffuse deficits is characteristic [1]. The most common cause of dementia is Alzheimer’s Disease (AD), which contributes to 60-80% of cases. The number of persons with AD is expected to increase and escalate dramatically in the coming years with aging of the baby boom generation. In 2019, there were an estimated 5.5 million Americans living with AD (https://www.nia.nih.gov/health/alzheimers-disease-fact-sheet). This number is projected to reach 13.8 million in the year of 2050 [2]. Apart from AD, dementia can also be caused by Alzheimer’s disease-related dementia (e.g., vascular dementia, Lewy body dementia, frontotemporal dementia, and Parkinson-related dementia).

Low power transcranial near - infrared (tNIR) light emitting diodes (LED) illuminate light which is outside of visible spectrum of human eyes but can efficiently penetrate skin and skull to reach brain parenchyma [3-5]. Recent biomedical reports indicate that low emission of tNIR light not only can be beneficial to various acute and chronic pathologic brain conditions, but also can help to maintain a healthy brain state. There are several molecular mechanisms how tNIR light stimulation can achieve positive therapeutic effects in brain tissue. One of the widely studied and recognized mechanism of action is the stimulation of mitochondria by photons and the consequent increase in intracellular production of adenosine triphosphate (ATP). This contributes to overcome the low ATP level associated with many neurological disorders [6-9]. Low power tNIR can also increase oxygenation, improve regional circulation and nutritional supplementation to the brain parenchyma by triggering nitric oxide (NO) production, which is an effective vasodilator [10-13]. The tNIR light exerts anti-inflammatory function through modulation of NF-κB system [14], tumor necrosis factor (TNFα), and beneficial regulation of other pro- and anti-inflammatory cytokines in brain parenchyma [15]. Through complex regulation of signaling molecules and TCF/LEF transcription factors [16-18], tNIR light activates anti-apoptotic, and anti-senescence cascades [17-19], and further exerts neuroprotective effects on both healthy and impaired brain cells and tissue [15]. The tNIR light can also promote synaptogenesis and neurogenesis through activation of brain derived neurotrophic factor (BDNF), which contributes to the infrastructure of brain function through environmental support by promoting new synaptic and neuronal growth [20, 21]. Reactive oxygen species (ROS) impair brain function and attribute to different neurological diseases [22, 23]. Photobio-modulation demonstrated effective regulation of cytokine-induced nitric oxide synthase production which helps to manage amount of ROS intracellularly and control oxidative stress and damage associated with it [24, 25]. In addition, photons in low power tNIR light carry low energy and deliver low power density, which makes it safe and incapable of heating or burning exposed tissues even with prolong and direct exposures [26].

In this study we examined the safety and potential therapeutic benefits of tNIR light stimulation in the treatment of patients with dementia.

## MATERIALS AND METHODS

This placebo controlled, randomized, double-blinded study was approved by institutional IRB, performed under protection embodied in the Basic Principles of the Declaration of Helsinki, and conducted at Baylor Scott & White Health (BSWH) Medical Center in Temple, TX. Sixty patients diagnosed with early and moderate dementia were enrolled and randomized to the active arm or control arm at a 2:1 ratio. Subjects, family caregivers, and investigators were masked. Inclusion criteria: 1) patients of all sex, age 50-85-year-old; 2) diagnosed with early- to mid-stage dementia or dementia-related symptoms; 3) generally healthy as indicated by recent physical examination within the last 6 months. Exclusion criteria: 1) diagnosed with actively growing, or a history of recurrent intracranial neoplasms; 2) history of epilepsy; 3) history of acute ischemic/hemorrhagic stroke.

Both active and sham light treatment helmet devices had 12 cranial modules with 70 LEDs/module and 2 foldable eye modules with 14 LEDs/module. Sham helmet devices designed identical to active devices but did not emit NIR light. Active helmet devices emitted low power NIR light with wavelength of 1060-1080nm and 15,000mW, irradiance or power density= 23.1mW/cm^2^, ~650cm^2^ per treatment area. Treatment protocol was two 6 minutes sessions daily for 8 consequent weeks at home with either active or sham device self-administered by patient/family caregiver.

Neuropsychological assessments of behavior, mood and cognitive performance were conducted at beginning (before the treatment) and at the end (within one day after the last treatment session) of study. Alzheimer’s Disease Neuroimaging Initiative (ADNI) neuropsychological battery was implemented to assess cognitive function of participants. ADNI battery included: 1) mini-mental state exam (MMSE) - a cognitive screener that briefly measures orientation, word recall, attention, working memory, copying skills, and abilities; 2) ADAS-Cog (including evaluation of spoken language, word finding ability and ability to comprehend, word recall, word recognition, and number cancellation test); 3) clock drawing test (CDT) - subjects asked to draw a clock to a requested time; 4) logical memory (immediate) - subjects asked to recall a story immediately after it is been read; 5) auditory verbal learning test- immediate (A.V.L.T.-1) - subjects asked to memorize and recall immediately list of words presented verbally in several trials; 6) digit span forward and backward (DSF and DSB) - subjects asked to recall given different sequences of numbers in same order (forward span) or reverse (backward span).; 7) category fluency test - subjects asked to name words in given category within one minute; 8) trail making tests A and B - subjects asked to connect circles in numerical and mixed (numerical-alphabetical) order, respectively in limited time; 9) WAIS-R digit symbol substitution test - a cognitive test to assess visual motor coordination, motor persistence, attention and response speed; 10) Boston naming test - subjects asked to name 30 objects/items printed in the book; 11) logical memory (delayed) - subjects asked to recall a story after 30 min delay; 12) auditory verbal learning test- delayed (A.V.L.T.-2) - subjects asked to recall list of words presented verbally 30 min prior the recording in several trials. Daily subjective responses record was documented by caregivers. Sleep associated findings were collected from caregivers’ daily logs and feedback notes from patients/caregivers during assessment visits. All assessments/evaluations were performed by the same examiner using same procedures across the study.

Analysis of variance (one-way ANOVA) was used to assess mean differences between testing occasions and analyze outcomes inside of each group. Clock drawing and clock copying tests were scored using Shulman method. Analyzed data were considered to be statistically significant when *p* < .05.

## RESULTS

Total of 60 subjects were enrolled into the study. Three subjects withdrew from the study due to health issues unrelated to the use of tNIR light therapy. Fifty-seven enrolled patients successfully completed the 8 weeks study course. Mean age of overall study population was 74.2 ± 7.7 years old with 60% of male and 40% of female distribution. Mean age of population treated with active device was 72.4 ± 8.2 years old with 59% of male and 41% of female distribution. Mean age of population treated with sham device was 77.8 ± 5.2 years old with 53% of male and 47% of female. Patients had no health or adverse effects reported during or after completion of study associated with use of tNIR light stimulation. Notably, both patients and family caregivers from the active arm shared positive feedback of noticeable changes in cognition and improved daily routine activities.

In the active arm, some patients appreciated longer and more peaceful night sleeps. Duration of sleep increased by 1 hour in average after 8 ± 2 days of the treatment. Recurring nightmares ceased in some patients receiving tNIR light therapy. Patients reported being more energetic, physically and mentally engaged in daily living. Caregivers noted that patients had less anxiety, improved mood, energy, and positive daily routine after approximately 14-21 days of treatment, which was not noted by caregivers in the placebo arm.

When results of active tNIR light treatments were compared with placebo effects in the end of trial, several improvements were noted as follows:

Mini-Mental State Exam (MMSE). In the active arm, the average MMSE score improved from 22.8 ± 2.6 at the beginning of treatment to 27.6 ± 2.8 (*p* < .001) at the end of the treatment, which was 4.8 points improvement (21.0% increase) over the course of treatment. In the control arm, the average MMSE score changed from 23.2 ± 1.6 at the beginning of treatment to 24.6 ± 2.5 (*p* = .066) at the end of the treatment, which was 1.4 points improvement (6.2% increase) over the course of treatment (Table 1).

**Table 1:** Study demographics, Mini Mental State Exam, Clock Drawing and Copying Tests, and Logical Memory Test I and II.

Characteristics	Sham Treatment					Active Treatment				
	Before		After			Before		After		
	Mean	S.D.	Mean	S.D.	*p -* value	Mean	S.D.	Mean	S.D.	*p -* value
MMSE	23.2	1.6	24.6	2.5	.06596	22.8	2.6	27.6	2.8	4.9E-11***
CDT	75.0	27.8	76.3	27.5	.89919	69.5	29.3	83.7	23.2	.08669
CCT	95.0	15.5	93.8	20.3	.84604	84.7	25.2	92.1	18.9	.15389
LMT-I	7.4	4.9	6.0	4.5	.39916	8.5	5.9	11.8	7.0	.03070*
LMT-II	4.3	4.6	2.7	4.0	.29372	6.5	5.9	9.5	7.8	.06417

MMSE: Mini-Mental State Exam. CDT: Clock Drawing Test. CCT: Clock Copying Test. LMT-I: Logical Memory Test - Immediate total story unit recall. LMT-II: Logical Memory Test - Delayed total story unit recall. S.D.: standard deviation * - *p* value < .05; ** - *p* value < .01; *** - *p* value < .001

**Figure 1.**
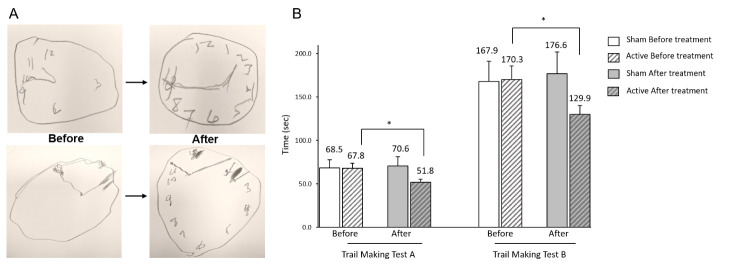
Cognitive improvements after treatment with t-NIR light twice a day for 8 consecutive weeks. (A) Clock Drawing Test. Figure demonstrate representatives of clock drawing tests performed by two different patients with dementia. Arrows indicate transition from results before to results after the course of the treatment. (B) Trail Making Test A and B. * - stands for statistically significant result with *p* < 0.05.

Clock Drawing Test (CDT). Positive trends of improved CDT scores were recorded in patients received active tNIR light treatment with mean value going up from 3.5 ± 1.5 at the beginning of treatment to 4.2 ± 1.2 (*p* = .08) at the end of the treatment. This was 0.7 points improvement (20.5% increase) over the course of treatment. Patients with sham treatment did not demonstrate significant changes in CDT with average scores shifting from 3.75 ± 1.39 to 3.81 ± 1.38 (*p* = .89), which is a mere 1.7% change in mean CDT score over the course of sham treatment (Fig. 1A, Table 1).

Clock Copying Test (CCT). Subjects with active tNIR light treatment showed trend of mean CCT score value improvement from 4.2 ± 1.3 at the beginning of treatment to 4.6 ± 0.9 (*p* = .15) at the end of the treatment resulted in 8.7% increase over the course of treatment. Subjects treated with sham devices demonstrated decrease of CCT performance from an average of 4.8 ± 0.8 at the beginning to 4.7 ± 1.0 (*p* = .84) at the end of the treatment (Table 1).

Logical Memory Test- immediate recall (LMT-I). Total story passage recall/learning trial in patients with active tNIR light treatment demonstrated improved performance. Average story recall scores increased significantly from 8.5 ± 5.9 at the beginning of treatment to 11.8 ± 7.0 (*p* = .03) at the end of the treatment and resulted in 3.3 points increase over the course of treatment. In comparison, placebo effect on execution of logical memory- I test demonstrated decrease in performance of story recall with average score shifting from 7.4 ± 5.0 at the beginning to 6.0 ± 4.5 (*p* = .39) at the end of the treatment. This resulted in 1.4 points decrease over the course of treatment (Table 1).

Logical Memory Test- delayed recall (LMT-II). Active device treated group also demonstrated improved performance by increase of average score by 3.0 points from 6.5 ± 5.9 at the beginning of treatment to 9.5 ± 7.8 (*p* = .06) at the end of the treatment. Sham treated group demonstrated decrease in performance similar to LMT-I test and resulted in test score decrease by 1.6 points from 4.3 ± 4.6 to 2.7 ± 4.0 (*p* = .29) over the course of treatment (Table 1).

Trail Making Test A. Positive results demonstrated by patients treated with active tNIR light device. They could perform noticeably faster and stayed focused on given task without being distracted or reminded with instructions. This test resulted in increased speed of performance, and the average time to completion of task decreased from 67.8 ± 36.5 seconds at the beginning of the treatment to 51.8 ± 22.7 seconds (*p* = .03) at the end of the treatment which was 16.0 seconds (23.5%) faster than the average performance before the treatment. This finding is in stark contrast to sham treated patients’ performance which demonstrated a broad range of responses from no change to slower performance. Average time to completion of sham treated dyad changed from 68.4 ± 35.5 seconds at the beginning of treatment to 70.6 ± 41.4 seconds (*p* = .87) at the end of treatment. This resulted in average of 2.2 seconds (6%) slower performance for the task when compared to results before sham treatment (Fig. 1B).

Trail Making Test B. More comprehensive and demanding in execution than Test A, Test B utilizes the same cognitive skills as Test A, but incorporates a mental flexibility component, and is given twice as much time for execution. Study patients undergoing active tNIR light treatment could successfully replicate Test A trends on this test. The average time to completion of task decreased from 170.3 ± 82.7 seconds at the beginning to 129.9 ± 55.3 seconds (*p* = .03) at the end of treatment, which is 40.4 sec (23.7%) faster performance compared to the speed of execution prior to the treatment. On other hand, sham treated patients, once again, demonstrated trend of slower execution after completion of treatment course. In this group, an average time of completion changed from 167.9 ± 80.8 seconds to 176.6 ± 88.0 seconds (*p* = .80) resulted in slower performance by 8.7 sec (5.2%) when compared with time of performance before the treatment (Fig. 1B).

Boston Naming Test (BNT). Patients treated with active device had significantly better average performance on BNT compared to sham treated group. Average score in this group improved from 24.4 ± 4.4 at the beginning to 26.6 ± 3.8 (*p* = .02) at the end of treatment, which is 2.2 points increase (8.8%) compared to levels before treatment. Some responses to the treatment resulted in up to 35.3% of score improvement. These results were noted in three cases in patients diagnosed with moderate dementia. Sham treated patients demonstrated insignificant BNT average score change from 23.4 ± 4.6 to 24.3 ± 4.9 (*p* = .58), which is 0.9 points shift (4.0%) compared to the level before treatment.

Digit Span Forward (DSF) and Digit Span Backward (DSB) Tests. Active device treatment resulted in positive change of DSF test average score from 6.9 ± 2.2 at the beginning to 7.6 ± 2.2 (*p* = .15) at the end of treatment, resulted in 0.7 points (10.3%) increase. In the active group, DSB test average score shifted from 4.8 ± 1.7 to 5.5 ± 2.1 (*p* = .17), a 0.6 point (12.4%) increase over the course of treatment. In the control group, neither DSF nor DSB had significant change in test performance compared to levels prior the treatment. Average test scores shifted from 6.4 ± 1.9 to 6.4 ± 2.0 (*p* = .92) and from 5.0 ± 1.4 to 5.0 ± 1.2 (*p* = 1.0) for DSF and DSB tests, respectively.

WAIS-R Digit Symbol Substitution Test. Treatment with active device resulted in trends of improvement of performance from 29.9 ± 11.4 to 33.7 ± 12.5 (*p* = .19), which is 3.8 (12.6%) points higher than before the treatment. Placebo effect did not trigger significant improvements of performance on this test and resulted in change of average score from 29.25 ± 13.9 to 29.8 ± 14.8 (*p* = .91), which is a mere 0.6 (1.9%) points change over the course of treatment.

Word Fluency Test. Both treated groups performed similarly with an average score change from 20.3 ± 7.3 to 22.9 ± 7.6 (*p* = .14) in the active arm and from 17.6 ± 6.6 to 19.1 ± 8.1 (*p* = .56) in the control arm, respectively.

Auditory Verbal Learning Test - Immediate (A.V.L.T. - 1). Treatment with active device resulted in statistically significant positive cognitive improvement in patients’ performance during the course of treatment. Immediate A.V.L.T.-1 test resulted in improvement of evaluated performance for Trial 1 by 48.6% (*p* < .001), Trial 5 by 31.2% (*p* = .001), Trial 1-5 Sum by 33.9% (*p* < .001), and Trial 7 by 47.7% (*p* = .002). On other hand, sham treated patients did not demonstrate statistically significant results in the end of the treatment. In most subtests, response to sham treatment had no change in performance (for Trial 1 and Trial 5) or insignificant trend (Trial 1-5 Sum) of improvement (Table 2).

Auditory Verbal Learning Test - Delayed (A.V.L.T. - 2). Delayed (30 min) recall and recognition subtests were evaluated in patients from both groups. Significant improvement was noted in the active group in delayed recall subtest with an average score improvement by 2.2 (63.5%) points increasing from 3.4 ± 2.9 to 5.6 ± 4.4 (*p* = .015) over the course of treatment. Significant observation was also noted for recognition subtest with performance improvement by 14.5% (*p* = .05) compared to score at the beginning of the treatment. However, sham treated patients did not demonstrate significant improvements on aforementioned subtests (Table 2).

**Table 2:** Auditory Verbal Learning Test - Immediate and Delayed Subsets.

Subtests	Sham Treatment					Active Treatment				
	Before		After			Before		After		
	Mean	S.D.	Mean	S.D.	*p -* value	Mean	S.D.	Mean	S.D.	*p -* value
A.V.L.T. - 1											
Trial 1	2.6	1.5	2.6	1.3	1	2.9	1.4	4.4	1.8	.00031***
Trial 5	5.6	2.4	5.6	2.5	1	7.2	2.8	9.4	3.0	.0015**
Sum 1-5	21.3	10.6	22.6	9.3	.69	26.7	9.9	35.7	12.2	.00078***
Trial 7	2.8	2.4	3.6	3.1	.41	4.6	2.6	6.8	3.3	.0022**
A.V.L.T. - 2											
Delay	1.0	2.1	2.0	3.3	.31	3.4	2.9	5.6	4.4	.015*
Recognition	6.5	4.6	7.3	4.5	.62	9.7	3.4	11.1	2.9	.058

A.V.L.T. - 1: Auditory Verbal Learning Test - Immediate. A.V.L.T. - 2: Auditory Verbal Learning Test - Delayed (30 min). S.D.: standard deviation * - *p* value < .05; ** - *p* value < .01; *** - *p* value < .001

## DISCUSSION

Our study successfully demonstrated home-implemented tNIR light stimulation for the treatment of dementia. This twice daily, eight weeks tNIR light sessions were self-administered by an older adult’s study population (74.2 ± 7.7 years old enrolled patients with dementia) with ease in the friendly environment of home and minimal instructions. This approach indicates that such technology can be used for a long period of time remotely by self-administration or with the help of a family or friend caregiver. A daily log was provided to patients and caregivers to impose treatment adherence and compliance. Portable and cushioned cases were provided along with the helmet device to ensure safe storage of the device and offer portability, increasing treatment accessibility and minimizing the potential conflict between treatment schedule and unexpected life events (e.g., travelling). These positive aspects of the treatment were represented in feedback from participants and their family caregivers. In comparison, other commonly used medical devices could be either invasive, such as a cardiac pacemaker, or require long hours continuous use causing discomfort, such as a continuous positive airway pressure (CPAP) machine. This demonstrates the feasibility and acceptability of a light emitting device, suggesting that the device can be a part of normal living in patients with dementia who need prolonged, continuous, and uninterrupted treatment without compromising quality of life.

Our study demonstrated safety of 1060-1080nm emitting spectrum, low power near infrared light used twice daily in this trial. This study is a second tNIR light treatment trial completed in Baylor Scott and White Health following a smaller recent pilot safety study using same technology and safety protocol, which was successfully conducted and completed in collaboration with Dr. Marvin Berman and his group from Quiet Mind Foundation, Elkins Park, PA, USA [27]. The two successfully completed trials serve as a good evidence in regard to the safety of the therapeutic protocol implemented in these trials, which is twice daily 6-min long non-invasive transcranial near infrared light stimulation. Considering reported safety in this and other clinical studies using NIR technology, more clinical studies are warranted using this technology and non-invasive route of NIR administration to investigate its full potential in the treatment of other neurological and neurodegenerative disorders.

As we look at the subtests from different individuals, the outcomes are even more exciting over only 8-week of active treatment: a participant with moderate dementia (MMSE = 16 prior to treatment) achieved a 75% improvement (MMSE = 28 at the end of the treatment); another patient had 80% improvement in the clock drawing tests (Schulman method scoring); two patients had 60% and 80% performance improvement in the clock copying test; two patients had 12 points (48%, from 7 to 19, total score 25) and 13 points (52%, from 7 to 20, total score 25) improvement in logical memory immediate and delayed subtests; executive function has improved by up to 73% in some patients evidenced by both trail making test A and B, and verbal learning and memory tests have improved by up to 35% as shown in the active verbal learning immediate and delayed tests. In general, across multiple subtests, patients in the group treated with active tNIR light emitting device demonstrated considerable improvement while patients in the sham treated group did not. These observed positive outcomes suggest beneficial effects of tNIR light stimulation on degenerative progression of neuropathological conditions associated with dementia. In sham treated group, natural progression of disease during the course of the study contributed to no positive change or decline in performance outcomes.

In the LMT-I, LMT-II, A.V.L.T.-1, A.V.L.T.-2 subtests and trail making test A, the pre-treatment mean values in the sham treated group appeared to be lower than pre-treatment mean values in the active group. However, the pre-treatment means values for sham treated group were higher in MMSE, CDT, CCT, DSB and trail making test B assessments. This could be attributed to the difference in mean age of study population in active (72.4 ± 8.2 y.o.) and sham (77.8 ± 5.2 y.o.) groups, to privilege of male population in active (59%) versus sham (53%), to relatively small sample size in the control group (n=17) versus active group (n=40), as well as explained by the underlying neuropathology. In addition, inherent ceiling and flooring effects could limit the reliability of these subtests. Nonetheless, further investigations with larger sample size, contribution of sex, age and causing neuropathology are warranted.

Our study also demonstrated improvement of sleep-in active group compared to feedback from patients and family caregivers in the sham group. Although without any specific measurement, it carries scientific merit and should be integrated into future studies given the important role of long and uninterrupted sleep-in dementia and elderly population, as sleep disturbance has been well demonstrated in dementia patients [28]. Caregiver notes and patient feedback indicated that patients treated with active tNIR light devices have improved night sleep with an average of 1 hour longer after 6-10 days of treatment. This was not reported in the control group. Patients in the active group also reported decreased episodes of recurrent nightmares they had for years before the study. These feedbacks are indicative of improved overall duration of night sleep which is impaired in patients with dementia [28]. This improvement alone could contribute to a better recovery. Other important findings were positive changes in daily routine, improved mood, less anxiety, more energy and engagement after approximately 14 to 21 days of treatment. These observations were shared by both the patients and family members. Great importance noted by many caregivers was improved patient’s engagement in daily living, helping around the household, remembering instructions and participation in activities. Further studies with specific measurements are warranted to examine patient-caregiver-family member relationship, activities of daily living with a focus on how tNIR light treatment can potentially mitigate family caregiver’s burden caring for patients with dementia.

Recent pre-clinical and clinical studies using tNIR light stimulation reported promising improvements in treatment of traumatic brain injury (TBI), Parkinson’s disease (PD), and Alzheimer’s disease (AD). Below we discuss latest findings and mechanisms of PBM in treatment of neuropathological conditions.

### Transcranial NIR light stimulation in treatment of traumatic brain injury.

A few studies have investigated the application of PBM in the treatment of TBIs. Some demonstrated successful treatment of TBI associated symptoms using red light stimulation [29, 30]. As discussed previously, there are several mechanisms known to be triggered by photons of NIR light which can reduce inflammation, constrain ROS damage, and activate ATP production in energy compromised brain area affected by TBI. Continuous production of ATP is required for successful coordination of metabolic, synaptic and immune efforts to consolidate the area of injury, facilitate recovery and manage local immune response [14, 15, 31-33]. Moreover, PBM- initiated neurogenesis and synaptogenesis promote reestablishment of axonal connectivity and rebuild the intrinsic nervous networks damaged in TBI [34].

With respect to TBI triggered inflammatory chain reaction and initiated generalized immune response, we support the idea that aforementioned pathological processes can be effectively addressed by implementing tNIR light therapy. This approach is supported by literature to be quite effective in acute and chronic TBI treatment, considering general safety and demonstrated efficacy of tNIR light stimulation *in-vitro*, *in-vivo* and growing number of recent human clinical trials in this field [29, 30]. One of the first clinical trials (small cohort study) using tNIR light stimulations to treat chronic and mild TBI reported positive improvements in cognition, behavior, and sleep of patients, but also suggested that more robust placebo-controlled studies are needed to ensure reliability of tNIR light in TBI treatment [30, 35].

### Transcranial NIR light in treatment of Parkinson’s disease.

Parkinson’s disease is a chronic CNS pathology caused by slow degeneration of substantia nigra (SN) pars compacta dopaminergic neurons. PD has various clinical presentations with motor and non-motor characteristics depending on the stage of disease. The most common symptoms are resting tremor, slowness of movements, rigidity of muscles, balance impairment, dementia, anxiety, depression, hallucinations and delusions. Recently, several studies have investigated the effects of tNIR light and PBM in treatment of PD using mice and monkey models [36-38]. Most commonly used disease-models were transgenic mice and 1-methyl-4-phenyl-1,2,3,6-tetrahydropyridine (MPTP) compound induced Parkinson’s model [39, 40]. In these studies, PBM has shown mostly neuroprotective and cell preserving effects by decreasing levels of hyper-phosphorylated tau and cellular degeneration caused by tau-protein in PD transgenic mice [40]. Low power LED laser treatment was effective in SN cells preservation in PD model [37]. In monkey PD model, after low dose red-light stimulation course have been observed improvement of facial expression, bradykinesia, and general activity [41]. An increased secretion of tyrosine hydroxylase in dopaminergic cells in response to red-light therapy or PBM stimulation might have attributed to the function improvement of SN cells in PD pathology [37, 38].

In this study, we have observed reduction of tremor with noticeable improvement of balance in patients with PD associated dementia. This could be consolidated with the improvement of micro-tremor observed during clock drawing test in this study (Figure 1A). More clinical trials are required to consolidate findings about benefits of tNIR light stimulations for treatment of patients with PD.

### Transcranial NIR light in treatment of Alzheimer’s disease.

Alzheimer’s disease is the most common neurodegenerative pathology of CNS with progressive memory loss and cognitive function impairment [41]. In AD, there are pathological buildup of toxic beta-amyloid peptide (β42) with hyper-phosphorylated tau proteins which result in formation of amyloid plaques and neurofibrillary tangles within brain tissue, respectively [42, 43]. Red light treatment can potentially benefit in management of AD through: 1) inhibition of apoptosis in AD targeted neurons [44]; 2) induction of resistance to neurotoxicity associated with toxic accumulation of β42 peptide [20, 45, 46]; 3) reduction of amyloid plaques formation in the brain parenchyma [47]. Recent clinical study has demonstrated significant improvement in MMSE scores, cognitive subscale behavior and mood subtests of AD patients treated with only daily intranasal applications of NIR light supplemented with only once a week transcranial NIR light session in duration of 12 weeks [48]. Even with a limited number of sessions using transcranial NIR light delivery could achieve positive improvements and align with some of our findings in this study. It is promising to construct more future clinical studies with transcranial applications of NIR light, which could potentially yield in more beneficial outcomes.

### Limitation of the study

Patient with mild dementia may have less room for improvement compared to patients diagnosed with moderate dementia. Additionally, some tests may not be powerful enough to detect fewer substantial changes. Therefore, robust changes in individual performances can be obscured by those with minimal changes and vice versa, when analyzing combined datasets from different stages of disease (different pathological states). This can potentially be addressed by stratifying patients’ data based on severity of dementia. Results of stratified patients’ data by stage of disease may represent a full potential of therapeutic range for each patient group.

### Conclusion

Transcranial applications in this study demonstrated safe and convenient approach to deliver NIR light to the brain without local or systemic side effects and adverse reactions. Treatment protocol designed for this trial was simple and successfully used at the convenience of home by elderly population. Observed positive cognitive, executive, and mood changes can benefit patients with dementia by improving quality of life and self-independence in daily lives, and thus helping their immediate family caregivers by decreasing their burden. More studies are necessary to look into family caregivers’ burden and to ensure reproducibility of positive findings.
